# A robust and reproducible automated MRI pipeline for quantifying tissue outcomes after experimental stroke in multi-center preclinical networks

**DOI:** 10.1162/IMAG.a.1328

**Published:** 2026-08-07

**Authors:** Kirsten M. Lynch, Ryan P. Cabeen, Andreia Lopes de Morais, Xuyan Jin, Erendiz Tarakci, Jessica Lamb, Basavaraju G. Sanganahalli, Jelena M. Mihailovic, Yamileck Olivas-Garcia, David B. Berry, Marcio A. Diniz, Joseph Mandeville, Fahmeed Hyder, Daniel R. Thedens, Ali Arbab, Shuning Huang, Adnan Bibic, Wyatt Austin, Bingren Hu, Mohammad B. Khan, Pradip K. Kamat, Arthur W. Toga, Patrick Lyden, Cenk Ayata

**Affiliations:** Laboratory of Neuro Imaging, USC Mark and Mary Stevens Institute for Neuroimaging and Informatics, Keck School of Medicine of USC, University of Southern California, Los Angeles, CA, United States; Department of Radiology, Massachusetts General Hospital, Harvard Medical School, Charlestown, MA, United States; Department of Physiology and Neuroscience, Zilkha Neurogenetic Institute of the Keck School of Medicine of USC, Los Angeles, CA, United States; Department of Radiology and Biomedical Imaging, Yale University, New Haven, CT, United States; Department of Emergency Medicine, University of California, San Diego, La Jolla, CA, United States; Department of Orthopaedic Surgery, University of California, San Diego, La Jolla, CA, United States; Icahn School of Medicine at Mount Sinai, New York, NY, United States; Department of Biomedical Engineering, Yale University, New Haven, CT, United States; Department of Radiology, University of Iowa, Iowa City, IA, United States; Department of Biochemistry and Molecular Biology, Medical College of Georgia at Augusta University, Augusta, GA, United States; Georgia Cancer Center, Augusta University, Augusta, CA, United States; Department of Diagnostic and Interventional Imaging, The University of Texas McGovern Medical School at Houston, Houston, TX, United States; F.M. Kirby Research Center for Functional Brain Imaging, Kennedy Krieger Research Institute, Baltimore, MD, United States; Russell H. Morgan Department of Radiology and Radiological Science, Johns Hopkins University School of Medicine, Baltimore, MD, United States; Department of Radiology, Duke University Medical Center, Durham, NC, United States; Department of Neurology, Medical College of Georgia at Augusta University, Augusta, GA, United States; Department of Neurology, Keck School of Medicine of USC, Los Angeles, CA, United States; Department of Neurology, Massachusetts General Hospital, Harvard Medical School, Charlestown, MA, United States

**Keywords:** ischemic stroke, preclinical trial, image analysis pipeline, lesion segmentation, rodent MRI, harmonization, deep learning, skull stripping

## Abstract

The failure to translate promising preclinical stroke therapies into clinical success is a multi-faceted problem; however, a critical contributing factor is the lack of rigorous, reproducible preclinical outcome measures. While magnetic resonance imaging (MRI) offers a translational alternative to traditional histology, its use in large, multi-site trials is challenged by data heterogeneity and the need for scalable analysis. To address this, we developed and validated a fully automated, open-source image analysis pipeline for the Stroke Preclinical Assessment Network (SPAN), a six-center preclinical trial network. The pipeline processed T2-weighted and apparent diffusion coefficient (ADC) maps from over 2,000 mice and rats, incorporating steps for cross-site data harmonization, deep learning-based brain extraction, and rule-based segmentation to quantify infarct volume, brain swelling, and atrophy. The pipeline demonstrated high accuracy, as automated lesion volumes strongly correlated with manual expert tracing on both MRI (R = 0.96) and 2,3,5-triphenyl-tetrazolium chloride (TTC)-stained tissue (R = 0.86). The U-net model for brain extraction achieved a Dice score of 0.96, and our harmonization method successfully reduced inter-site variability in quantitative MRI parameters. This robust and reproducible pipeline provides a scalable framework for standardizing tissue outcome assessment, enhancing the rigor of multi-site preclinical studies.

## 1 Introduction

Ischemic stroke is a leading cause of death and disability worldwide (Saini et al., 2021), yet despite over a thousand interventions showing promise in preclinical animal studies, these successes have so far failed to translate into effective clinical therapies (Bosetti et al., 2017). This translational failure is attributed to many factors, including a lack of rigor, reliability, and reproducibility in underpowered, single-laboratory studies (Sena et al., 2010; van der Worp et al., 2010; Voelkl et al., 2018). To address pre-clinical rigor and reproducibility, the NIH-sponsored Stroke Preclinical Assessment Network (SPAN) was launched as a multicenter preclinical trial network that implements robust clinical trial methodologies, including centralized randomization, blinding, and strict adherence to standard operating procedures (Lyden et al., 2022). In the largest pre-clinical trial of its kind, SPAN tested six promising interventions in a large cohort of mice and rats using transient endovascular filament occlusion of the middle cerebral artery (MCAO) as a model for ischemic stroke (Lyden et al., 2023). The primary efficacy endpoint was a behavioral test, as required for clinical qualification by regulatory bodies. To provide an additional and direct, objective measure of injury, SPAN included tissue outcome as a complementary secondary endpoint.

Historically, preclinical stroke studies measure tissue outcomes with 2,3,5-triphenyl-tetrazolium chloride (TTC) staining or histology or both (Cole et al., 1990; Zille et al., 2012). While common, these methods have critical weaknesses: TTC staining intensity is often variable, manual slicing introduces tissue distortion, and outlining the gradual transition from infarct to normal tissue is challenging, leading to low interrater reproducibility (Friedländer et al., 2017). Furthermore, both TTC and histology are terminal, allowing for only a single time point assessment per animal, which prevents the longitudinal study of lesion evolution and requires more animals. In contrast, magnetic resonance imaging (MRI) is a non-invasive alternative that overcomes these limitations. MRI provides readouts identical to those used in human trials, offering a more direct translational path. MRI preserves whole-brain anatomy *in situ* and can be repeated in the same animal over time, enabling longitudinal assessment of biological changes like edema and atrophy while improving reproducibility. Because standardization can be achieved across different scanners and lends itself well to a multicenter study design, SPAN employed MRI as a state-of-the-art and clinically relevant tool for assessing tissue outcome.

Algorithms have been proposed for MRI-based stroke imaging (J. An et al., 2023; Chang et al., 2021; Mulder et al., 2017), demonstrating the feasibility of MRI in preclinical stroke studies. However, scaling these methods to meet the demands of a high-volume, high-throughput, multicenter network like SPAN introduces unique challenges, such as differences in scanner hardware, radiofrequency coils, and *in vivo* imaging environments, all of which require reconciliation to ensure data consistency. Changes in equipment and personnel, as well as occasional breakdowns, must be accommodated over time. More importantly, a fully automated and robust analysis pipeline with novel computational tools is needed to process thousands of MRIs, which could survive real-world challenges for universal adoption.

Here, we report on a fully automated analysis pipeline for image-based stroke assessment implemented in SPAN to provide robust processing and continuous reporting of data from multiple time points after injury at multiple imaging centers. This work provides an end-to-end solution that not only quantifies lesion volume but also measures brain atrophy, ventricular volume, and midline shift in two rodent species. Our pipeline is specifically designed to handle the complexities of multi-site longitudinal data and has been validated on a dataset of over 2,000 animals, making it the largest preclinical stroke MRI study.

## 2 Methods

### 2.1 SPAN structure, stroke model, and study population

The details of SPAN structure, stroke model, and standard operating procedures (SOP) have been previously published (Lyden et al., 2022, 2023; Morais et al., 2023, 2024). Experiments and data collection were performed across six academic research laboratories [Johns Hopkins University (JH), University of Iowa (IW), Yale University (YL), Massachusetts General Hospital (MG), University of Texas McGovern Medical School at Houston (UT), and Augusta University (AU)], and centralized management of enrollment and treatment allocation was carried out by a coordinating center (CC) at the University of Southern California (USC). Before beginning any work, this protocol was approved by the IACUC at each of the participating research laboratories. Focal ischemic stroke was induced using a 60-minute transient endovascular filament middle cerebral artery occlusion (MCAO). MRI was performed on days 2 and 30 after MCAO. To maximize translational rigor, the network-wide study design mandated the use of two species across all sites. Therefore, the study population included the modified intention-to-treat (mITT) cohort that survived surgery, with 1,772 mice, of which 1,452 (82%) had an MRI on day 2 and 1,055 (60%) had a second MRI on day 30, and 670 rats, of which 588 (88%) had an MRI on day 2 and 563 (84%) had a second MRI on day 30. These subjects were enrolled in four stages (Lyden et al., 2023).

### 2.2 SPAN MRI pipeline validation pilot

Before the trial, SPAN performed a pilot study to validate automated MRI lesion segmentation against the commonly used TTC staining. Pilot study enrolled a total of 36 mice at the 6 trial laboratories to ensure the data were representative of the variation in SPAN and the greater stroke field. Animals were sacrificed immediately after the day 2 MRI to perform TTC staining on coronal slices. Each laboratory photographed TTC-stained brain slices front and back, and the images were shared with the MRI team. This resulted in a total of 746 images of individual slices. We chose a total of six annotators (one from each SPAN site) to provide at least two annotations for each image. Due to the scale of the task and logistics of working with a distributed team, we built an online web-based annotation system based on LabelMe (Russell & Torralba, 2008) to support remote annotations. We modified LabelMe to use a restricted set of drawing operations, to anonymize the images and raters, to enable deployment across multiple isolated servers for each rater, and to provide instructions to label the brain, lesion, and any other relevant features. Once collected, we computed the total lesion volumes by extracting the 2D infarct area from each annotated slice. These areas were then summed across all slices for a given subject and multiplied by the standard brain matrix slice thickness (ranging 1–2 mm across sites) to reconstruct the total 3D infarct volume in mm³. This approach allowed for a direct, independent volumetric correlation against the 3D MRI-derived volumes, circumventing the need for complex and often inaccurate slice-by-slice coregistration between the ex vivo histological sections and the in vivo MRI scans. The MRI data from the 36 mice in the pilot were processed, and the lesion volume fraction was computed via the SPAN MRI pipeline, just like all the other trial subjects, without manual intervention.

### 2.3 SPAN MRI acquisition protocol

Table 1 summarizes the MRI hardware, software, and scan environment for each of the six imaging sites. Network sites had five horizontal-bore Bruker scanners and one General Electric scanner. Field strengths varied from 7T to 11.7T across sites (3 sites had 7T; 2 sites had 9.4T; 1 site had 11.7T). Since the participating sites used B0’s ranging from 7T to 11.7T, dependencies of T2 on field strength were addressed by the harmonization as described below. Scaling correction for R2 was applied at each field. All but one site used volume RF coils for transmission with surface RF coils for reception, and one used a surface RF coil as the transceiver. All sites had higher-order shims, which allowed magnetic field homogeneity to be optimized by localized shimming. Since retrospective correction of B1 field inhomogeneity may alter lesion intensity due to a lack of priors, we refrained from any permanent alteration of these data. Furthermore, the signal-to-noise ratio of the images from the surface coil was sufficiently high, and the T2 and ADC fit did not reflect the B1 field inhomogeneity in the fitted parameter maps.

**Table 1.** MRI facilities and scan environment at the study sites.

*Study sites*	JH	IW	YL	MG	UT	AU
*Brand*	Bruker	General Electric	Bruker	Bruker	Bruker	Bruker
*Magnet strength (T)*	11.7	7	9.4	9.4	7	7
*Bore size*	16 cm	21 cm	16 cm	11.7 cm	30 cm	20 cm
*Software*	ParaVision 6.01	Discovery MR901 v14.0	ParaVision 6.01	ParaVision 6.01	ParaVision 5.1	ParaVision 6.01
*Transmit coil*	volume	volume	surface	volume	volume	volume
*Receive coil*	4-channel surface	2-channel surface	surface	4-channel surface	surface	surface
*Fat sat?*	yes	yes	yes	yes	yes	yes
*Anesthesia*	Isoflurane	Isoflurane	Isoflurane	Isoflurane	Isoflurane	Isoflurane
*Inhalation gas*	70% air, 30% O_2_	70% N_2_O, 30% O_2_	70% N_2_O, 30% O_2_	70% air, 30% O_2_	70% N_2_O, 30% O_2_	70% N_2_O, 30% O_2_
*Temperature monitoring*	Yes	No	Yes	Yes	Yes	Yes
*Pulse oximetry*	No	No	Yes	Yes	No	No
*Respiratory monitoring*	Yes	Yes	Yes	Yes	Yes	Yes
*Mouse scan duration (min)*	40	40-50	30-40	30-35	25	45-50
*Rat scan duration (min)*	35	40	40-45	30	35-40	48
*Cost per hour ($), mouse*	180	120	150	300	100	100
*Cost per hour ($), rat*	185	120	280	325	100	100
*Number of mITT subjects*	410	434	404	400	406	389
*Number of MRI scans*	627	639	561	724	487	623
***Mouse** Day 2 SNR (Mean±SD)*	ADC: 4.8 ± 2.4 T2: 9.6 ± 3.8	ADC: 4.0 ± 0.5 T2: 7.3 ± 1.2	ADC: 5.1 ± 1.9 T2: 7.4 ± 3.4	ADC: 2.6 ± 0.4 T2: 4.6 ± 0.9	ADC: 9.0 ± 1.4 T2: 12.5 ± 2.0	ADC: 1.8 ± 0.2 T2: 4.4 ± 0.6
***Rat** Day 2 SNR (Mean±SD)*	ADC: 4.0 ± 0.5 T2: 5.7 ± 0.4	ADC: 3.2 ± 0.2 T2: 4.1 ± 0.5	ADC: 4.7 ± 0.4 T2: 5.6 ± 0.7	ADC: 3.7 ± 0.6 T2: 5.4 ± 0.7	ADC: 6.1 ± 0.6 T2: 6.6 ± 0.8	ADC: 3.0 ± 0.2 T2: 5.1 ± 0.4
***Mouse** Day 30 SNR (Mean±SD)*	ADC: 4.2 ± 2.0 T2: 8.7 ± 3.3	ADC: 4.3 ± 0.6 T2: 8.2 ± 1.0	ADC: 4.9 ± 1.9 T2: 7.0 ± 3.4	ADC: 2.5 ± 0.4 T2: 4.5 ± 0.9	ADC: 8.7 ± 1.1 T2: 12.3 ± 1.5	ADC: 1.7 ± 0.1 T2: 4.0 ± 0.6
***Rat** Day 30 SNR (Mean±SD)*	ADC: 3.8 ± 0.5 T2: 5.8 ± 0.5	ADC: 3.2 ± 0.5 T2: 4.1 ± 0.6	ADC: 4.4 ± 0.4 T2: 5.1 ± 0.4	ADC: 3.5 ± 0.9 T2: 5.4 ± 2.0	ADC: 5.2 ± 1.0 T2: 5.6 ± 1.2	ADC: 2.9 ± 0.1 T2: 5.0 ± 0.3
***Acquisition time (min)***	ADC: 9:36 T2: 9:36	ADC: 11:00 T2: 19:00	ADC: 9:30 T2: 6:30	ADC: 6:00 T2: 5:30	ADC: 6:54 T2: 9:36	ADC: 4:14 T2: 8:00

Johns Hopkins University (JH), University of Iowa (IW), Yale University (YL), Massachusetts General Hospital (MG), University of Texas McGovern Medical School at Houston (UT), and Augusta University (AU).

Animals were anesthetized using isoflurane (1.5–2.5%), freely breathing a mixture of 30% O_2_ and 70% N_2_O or room air through a nose cone and then placed in a custom-built MRI-compatible holder. Body temperature was monitored throughout the procedure using a rectal probe and maintained within 35–37°C using either warm water pumped through a pad or warm air blown across the subject’s body. Respiration was continuously monitored using an MRI-compatible respiratory system at all sites. Rather than recording numerical rates as an experimental variable, operators continuously adjusted the isoflurane anesthesia to maintain each animal’s respiration within normal, species-specific physiological ranges. Some sites used pulse oximetry to measure oxygen saturation.

The MRI scanning protocol aimed to maximize information gained per unit scan time and to enable automated lesion segmentation. We obtained quantitative transverse relaxation time (T2) and apparent diffusion coefficient (ADC) maps so that elevated T2 could be used to segment the ischemic lesion and cerebrospinal fluid (CSF) from normal tissue, and ADC could differentiate the lesion from CSF. T2 maps were obtained using a multi-slice multi-echo sequence for volume coil sites (TR = 4,500 ms, 10 echoes equally spaced from 10 to 100 ms), and sequential single echoes (15, 45, 75 ms) for the surface coil site to avoid biased signal variation in depth direction. The ADC map was acquired using a conventional spin-echo diffusion-weighted imaging (DWI) pulse sequence (TR/TE = 1,500/25 ms; b-values = 0, 500, 1,000 s/mm^2^ along the z-direction, δ = 4 ms, Δ = 12 ms). These multiparametric MRI sequences approximated those clinically used to evaluate acute ischemic stroke. In mice, the resulting field of view (FOV) was 19.2 mm in-plane and 15 mm in slice direction with 0.5 mm thickness (.15 × .15 × .5 mm voxel resolution). In rats, the resulting FOV was 25.6 mm in-plane and 24 mm in slice direction with 0.8 mm thickness (.2 × .2 × .8 mm voxel resolution). The matrix size was 128 × 128 in slice with 30 slices for both rats and mice. This configuration provided enough spatial resolution for lesion segmentation and morphometry while minimizing the scan times in both species.

Each animal was scanned 2 and 30 ± 2 days after MCAO using the above protocol. The day 2 scan primarily aimed to quantify the infarct volume and ischemic swelling, while the day 30 scan aimed to quantify tissue loss as a late reflection of ischemic injury. Baseline MRI scans prior to MCAO were not acquired in this cohort.

### 2.4 Central image upload and data management

The SPAN network implemented centralized data storage, archiving, indexing, and sharing. All imaging data were uploaded to the Imaging Data Archive (IDA) at the USC Laboratory of Neuro Imaging (LONI) (Crawford et al., 2016; Neu et al., 2023). Image data were encoded by the scanner and stored in the IDA in the DICOM format, which included metadata such as the scanning site, scanner operator, software versions, MRI sequence parameters, magnetic field strength, acquisition time, and experimental time point. Data were uploaded via a Java web tool (Crawford et al., 2016), requiring minimal user intervention and providing verification upon successful upload. Once uploaded, the data were accessible with fine-grained access controls, allowing authorized users to view and download image data in a variety of formats in batch mode.

### 2.5 Automated image processing and analysis pipeline

**Fig. 1.**
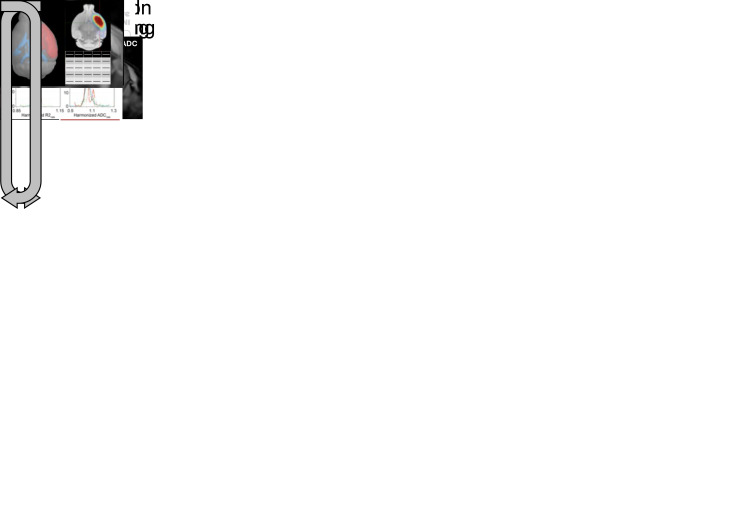
A schematic overview of our image analysis pipeline.

Our image analysis software pipeline (Fig. 1) was implemented with the Quantitative Imaging Toolkit (QIT) (Cabeen et al., 2018), Advanced Normalization Tools (ANTs) (Avants et al., 2009), and R 4.1.0 for plotting and statistical analysis. The pipeline includes image acquisition, preprocessing, quality assessment, harmonization, brain and lesion segmentation, midline shift quantification, and analytic reporting. Data were processed at the USC Neuro Imaging Computing Center (NICC) using a 4096-core Sun Grid Engine computing environment running CentOS 7. The pipeline is available to download online (https://github.com/cabeen/span-mri). It is designed to be compatible with all major operating systems and integrate into grid computing environments.

#### 2.5.1 Preprocessing and quality assessment

Scanners from different sites may produce data that differ systematically in small ways. Therefore, the first step in our pipeline was to standardize the image file format, names, and organization across all imaging sites. Data preprocessing included parsing DICOM tags, sorting by imaging parameters, fixing image coordinates, conversion to NIfTI format using dcm2niix (Li et al., 2016), and uniform tricubic resampling to 150 μm isotropic resolution. This specific resolution was chosen because it preserves the exact native in-plane resolution of the mouse scans (150 µm) and requires only minor in-plane upsampling for the rat scans (200 µm). Interpolating the anisotropic slice thickness (0.5 mm for mice and 0.8 mm for rats) into an isotropic grid was a strict methodological requirement to enable robust 3D atlas registration and to support our U-Net architecture, which relies on isotropic dimensions to accurately average 2D predictions across all three orthogonal image planes. We also applied adaptive non-local means denoising with voxel-wise noise estimation using a bandwidth set to 10% of the mean image intensity (Manjón et al., 2010). We generated reports of image quality metrics by segmenting foreground and background using Otsu thresholding (Otsu, 1979) and computing the signal-to-noise ratio, contrast-to-noise ratio, and signal variance-to-noise ratio. Specifically, SNR was computed on a per-scan basis by dividing the mean signal intensity of the brain foreground by the standard deviation of the background noise:

$$SNR=\;\frac{\mu_{foreground}}{\sigma_{background}}.$$

**Fig. 2.**
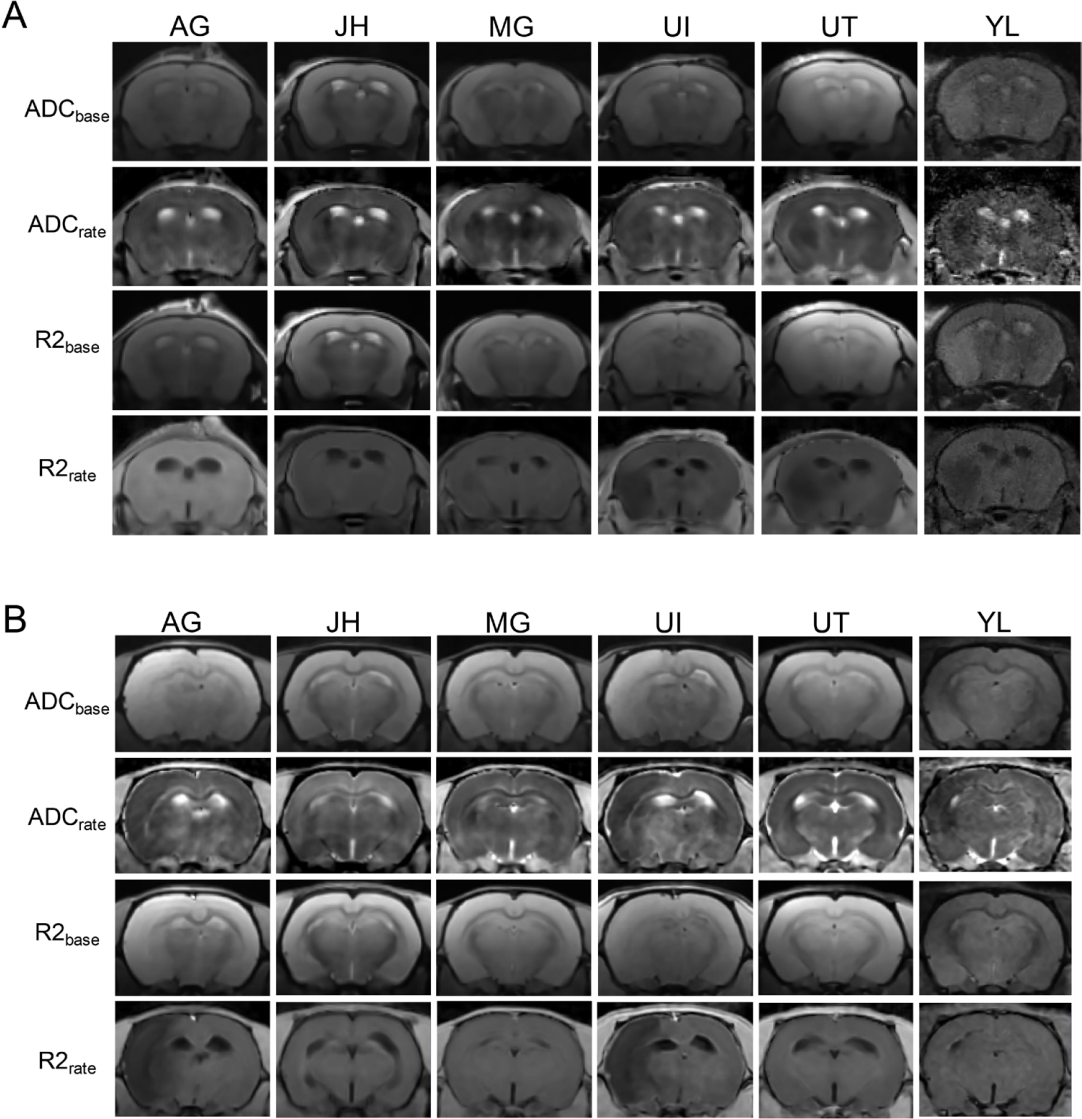
Example (A) mouse and (B) rat data showing imaging data from SPAN, which included four quantitative parameter maps collected at six distinct imaging centers. Johns Hopkins University (JH), University of Iowa (IW), Yale University (YL), Massachusetts General Hospital (MG), University of Texas McGovern Medical School at Houston (UT), and Augusta University (AU).

The background was derived as all voxels falling outside the Otsu-derived head mask. Because this calculation utilizes the entire head volume, rather than a localized region of interest in white matter, the resulting aggregate SNR values are highly conservative. We then performed relaxometry to derive quantitative parameter maps (mono-exponential model with baseline signal and decay rate parameters), and performed N4 bias field correction on the resulting parameter maps to correct for low-frequency intensity non-uniformity (Tustison et al., 2010). For simplicity of presentation, we report all T2 values as the inverse relaxation rate (R2). The derived quantitative measures included a signal baseline (ADC_base_, R2_base_) and rate of decay (ADC_rate_, R2_rate_) for the multi-echo T2 and DWI scans (Fig. 2).

We performed quality assurance on a rolling basis as data were received by inspecting image quality reports and summarizing the residuals of parameter fitting. The investigator overseeing the pipeline (RPC) alerted the CC of subjects with poor image quality, who alerted the site to see whether the technical issue could be rectified to recover the images. We created mosaic image plots for each case and parameter value to share and review the data qualitatively across the team.

#### 2.5.2 Brain extraction

Brain extraction was accomplished using two methods: a traditional rule-based algorithm that proved sufficient for rat brains, and a deep-learning U-net model that was developed to achieve the necessary robustness for mouse brains. Both methods are described below.

##### 2.5.2.1 Traditional rule-based segmentation approach

Our initial brain extraction stage of the SPAN pipeline used a traditional rule-based approach. First, a foreground extraction was performed with an Otsu threshold (Otsu, 1979), followed by histogram-based contrast enhancement and edge-preserving smoothing with a non-local means filter (Manjón et al., 2010). Gradient magnitude estimation was then performed with a Sobel filter bank, and the resulting image was thresholded and segmented using graph-based segmentation (Felzenszwalb & Huttenlocher, 2004). An initial brain mask was isolated by filtering the segmented image using a minimum region threshold of 3,000 voxels. The final brain mask was computed through iterative opening, closing, and filling of the mask, followed by regularization with a Markov random field (Felzenszwalb & Huttenlocher, 2006). This procedure was applied to the ADC_base_ contrast due to the minimized observed contrast between the lesion and normal appearing tissue. The traditional rule-based segmentation approach was able to sufficiently extract the brain mask in the vast majority of rats and was thus used as the final step for brain extraction in this species. The approach was effective in a large proportion of mouse cases (over 80%), and most failures were due to partial volume effects that blurred the skull boundary, resulting in either small errors on the superficial surface of the brain or catastrophic errors in which the brain mask leaked to the rest of the head. To preserve as much data as possible, successful cases from this approach were used to bootstrap a more accurate and modern approach for mice using U-nets.

##### 2.5.2.2 Modern neural network approach

We developed a brain extraction tool in mouse brains using a convolutional neural network approach with a U-net architecture (Ronneberger et al., 2015), which has demonstrated superior performance in previous rodent imaging studies (De Feo et al., 2021; Hsu et al., 2020). Our model was implemented in PyTorch and included 5 contraction/expansion stages, a kernel size of 64, and an input resolution of 128 x 128. We trained a single 2D U-net with slices from all three image planes, and during inference, we applied the 2D U-net three times to include every possible image plane. We finally took the average prediction from the three applications for each voxel to obtain the final prediction. We trained our model with the ADAM optimizer with a cross-entropy loss, a learning rate of 0.0001, a batch size of 20, and included albumentations-based data augmentation for translation, rotation, scaling, contrast, and deformations (Buslaev et al., 2020). Our system was a Lambda Labs workstation with Ubuntu 20.04, an AMD Ryzen Threadripper 3970X 32-Core CPU, and an Nvidia 1080 Ti 12 GB GPU. We created our training dataset from the best-performing results from the previously described rule-based approach. We selected training examples from 180 cases and hold-out testing and validation examples from 30 cases; each was split roughly evenly among the imaging centers and time points. We trained the model for 10 epochs on the training data, selected the best model with the validation data, and finally evaluated hold-out performance with the test data. We report our accuracy estimates with the Dice coefficient (Dice, 1945).

##### 2.5.2.3 Evaluation

SPAN provided a wide range of data types, varying across imaging contrasts, scanner hardware, and longitudinal time points. We investigated how a typical U-net architecture performs by training and evaluating a variety of experimental U-net models, which were fit to subsets of our data with the goal of understanding how performance varies across these different conditions. First, to evaluate performance across imaging contrasts, we trained models that included all four quantitative parameters (R2_base_, R2_rate_, ADC_base_, and ADC_rate_) as a multi-channel input or a single-channel model trained on the concatenated whole of these image contrasts. We also examined the performance of four models trained separately on each individual image contrast (i.e., “single contrast”). Next, to evaluate performance across sites, we compared the performance of the model using data from all six sites to a model generated by holding out data from half of the sites, which is meant to understand the generalizability of the model to unseen data, for example, from the new sites that might be added to SPAN in the future. The training and validation data were from three sites (JH, IW, YL), and the testing data were from the other three sites (AU, MG, UT). As a final evaluation step, we applied our brain extraction tool to the entirety of the SPAN stage 1 dataset (N = 1,368) and qualitatively assessed the results to determine whether they passed quality control; we report this outcome as the failure rate.

#### 2.5.3 Normalization

**Fig. 3.**
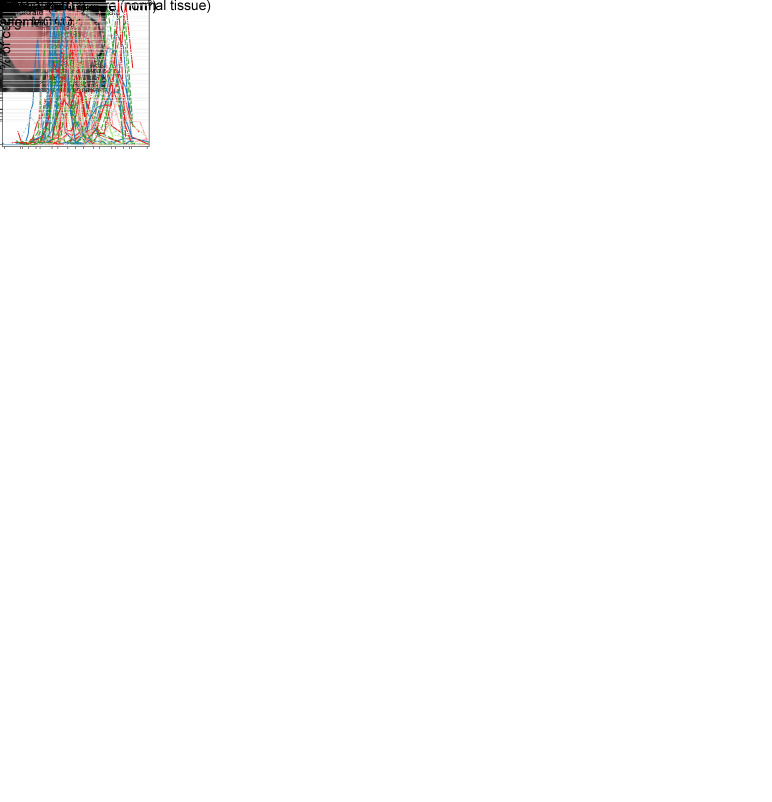
Volume normalization and harmonization of R2_rate_ and ADC_rate_ maps. (A) Representative images showing day 2 and day 30 mouse R2 and ADC maps and brain extraction using a well-established convolutional neural network approach using a U-Net architecture. (B) Polygon histograms showing raw and spatially normalized brain volume distributions (shown in pink in panel A) among the six sites represented by different colors. (C) Polygon histograms showing contralesional normal hemispheric R2 and ADC signal distributions among the six sites represented by different colors before and after harmonization (upper and lower rows, respectively). Site 1: AG, Site 2: UI, Site 3: JH, Site 4: MG, Site 5: UY, Site 6: YL.

Small, but significant differences in total intracranial volume were observed across the sites (Fig. 3B). Since all sites used the same mouse strains of a similar age, the differences were unlikely to be biological. Therefore, we normalized each site’s average intracranial volume to a global mean using a correction factor. All subsequent volumetric measurements utilized this scale correction.

#### 2.5.4 Harmonization

Quantitative image parameters (R2, ADC) of normal brain tissue (e.g., hemisphere contralateral to the arterial occlusion) differed substantially across sites (Fig. 3C), likely due to differences in hardware, scan technique, and magnetic field strengths ranging from 7T to 11.7T. To address this, we implemented a per-scan global intensity normalization procedure to harmonize the data. For each individual skill-stripped scan, a smoothed histogram of the intensity distribution was computed within the brain mask, and the peak value (i.e., the mode, M) was identified. The harmonized image, $I_{harmonized}\left(x\right)$, was then calculated by scaling the intensity I at each voxel x by this mode, effectively bringing the most likely value to 1:

$$I_{harmonized}\left(x\right)=\;\frac{I\left(x\right)}{M}.$$

The mode was chosen because it is less affected by distributional skew due to the presence of a lesion. The underlying assumption of this global intensity normalization approach is that the mode of the parameter distribution strictly corresponds to normal-appearing tissue, and this baseline should be matched across all cases. Because this scaling was applied on a per-scan basis rather than a per-site basis, it inherently corrected for individual scan fluctuations as well as systematic inter-site differences, including the physical dependencies of transfer relaxation rates on different B0 field strengths.

While a limitation of global intensity normalization is the assumption that the target tissue compartment remains relatively constant in volume and baseline signal, our qualitative inspection of the smoothed histograms confirmed its appropriateness. Furthermore, to quantitatively verify the efficacy of this harmonization, we measured the inter-site variance of the median normal tissue values. Following harmonization, the inter-site coefficient of variation (CoV) in the mouse cohort was reduced from 13.64% to 2.00% for R2_rate_, and from 16.09% to 1.99% for ADC_rate_. Similarly, in the rat cohort, the inter-site CoV was reduced from 11.67% to 1.23% for R2_rate_, and from 10.46% to 1.21% for ADC_rate_.

#### 2.5.5 Atlas-based registration

Following brain extraction, we performed rigid registration of each case to the Mouse BIRN Atlasing Toolkit (MBAT) brain atlas using the skull-stripped R2_rate_ parameter map and applied the transform to the rest of the native space parameter maps (Lee et al., 2010; Mackenzie-Graham et al., 2007). The alignment to the atlas facilitated consistent visualization of image slices and the transfer of anatomical priors.

#### 2.5.6 Tissue, ventricle, and lesion segmentation

**Fig. 4.**
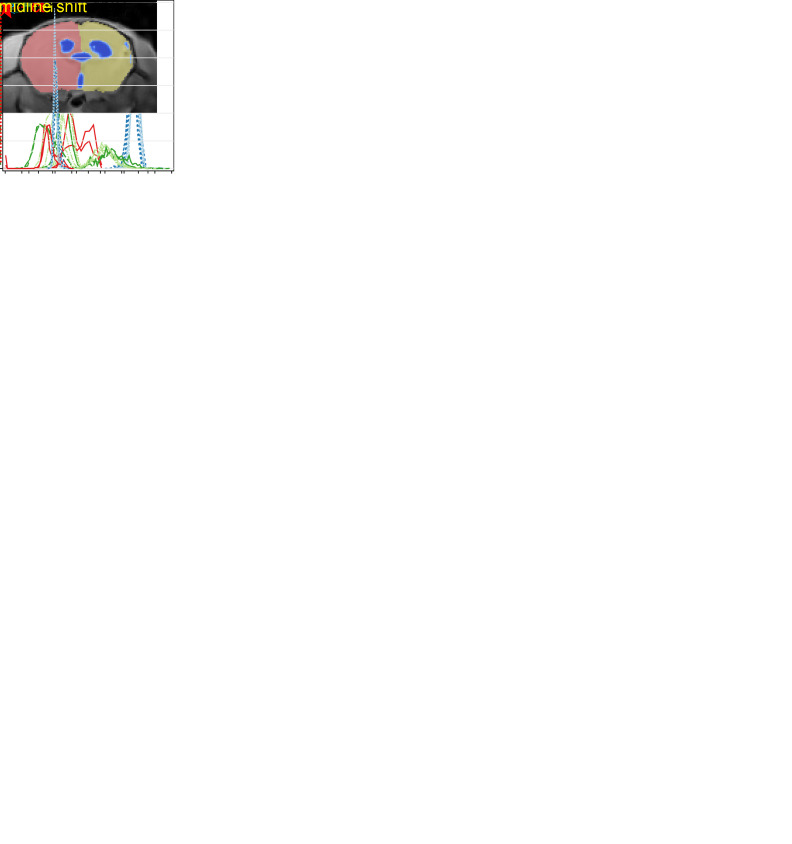
Infarct and normal tissue segmentation using R2_rate_ and ADC_rate_ thresholds. (A) Polygon histograms illustrate the harmonized ADC and R2 distribution on day 2 and day 30 MRIs within the infarct (red), ipsilateral and contralateral ventricles (CSF, green), and ipsilateral and contralateral normal brain tissue (nl, blue). Vertical dotted and dashed lines indicate the threshold values used to segment the infarct and CSF on the R2 and ADC maps, respectively. Asterisk shows the overlap in ADC values between the infarct and normal tissue, preventing us from using the ADC to delineate the infarct. Note that lesions were not quantified on day 30 due to the lack of a reliable indicator. (B) Representative images in mice display the segmented hemispheres, ventricles, and infarct for volume measurements on days 2 and 30 after MCAO. Calculated readouts include ischemic swelling, tissue loss (i.e., atrophy), and midline shift. C, contralateral; I, ipsilateral; m, midline; w, hemispheric width.

Lesion segmentation was performed using a multiple threshold image processing approach applied ADC and R2_rate_ parameters in standard atlas space. Prior to segmentation, all quantitative maps underwent N4 bias field correction and global intensity harmonization to ensure signal stability across sites. Because our harmonization process successfully aligned the signal intensity distributions across hardware, we utilized fixed segmentation thresholds across all sites, time points, and species. The specific thresholds of the pipeline were determined through an iterative process of review by the computational team and stroke experts who have deep knowledge of stroke pathophysiology and how those tissue changes are revealed by MRI. To choose the specific lesion segmentation threshold, we ran the pipeline with a parameter sweep across the full range of possible values. We then inspected both the resulting individual segmentation maps to find a threshold that ensured the region boundaries were acceptable to the stroke experts, paying close attention to the low and high end to ensure that small lesions and large lesions were accurately determined. (Fig. 4A).

The automated segmentation algorithm utilizes a sigmoid-based probability estimation, where each imaging modality (R2_rate_, ADC_rate_, and ADC_base_) is mapped to a [0,1] probability via a sigmoid function centered at the defined threshold value. An initial lesion map was first defined by applying an inverted sigmoidal soft threshold of 0.8 to the harmonized R2_rate_ map and a threshold of 1.5 to the harmonized ADC_rate_ map (Fig. 4A, vertical red dotted line). A similar procedure was used to segment CSF with an R2_rate_ threshold of 0.75 and ADC_rate_ threshold of 1.25 (not inverted) to provide a clear separation of the lesion and CSF (Fig. 4A, vertical green dashed lines). Hence, we identified the infarct as dark in both R2_rate_ and ADC_rate_, and CSF as dark in R2_rate_ and bright in ADC_rate_. The remainder of the brain was defined as normal-appearing tissue. To enforce multi-modal agreement, a joint probability is calculated as the product of the individual sequence probabilities. We then applied a two-threshold seed-and-grow strategy within standard atlas space: an initial high joint-probability threshold of 0.5 is used to identify core lesion seeds, which are subsequently expanded to a lower probability boundary of 0.45 using morphological dilation. Finally, hysteresis thresholding, mathematical morphology, and atlas-based exclusion were applied to remove any remaining false positives contralateral to the arterial occlusion. Harmonized R2 and ADC parameters from normal tissue and CSF were highly consistent between day 2 and day 30 scans (Fig. 4A, Day 30). Erroneous segmentations due to misregistration errors, motion artifacts, and incorrect field of view; such cases were excluded from further analysis.

#### 2.5.7 Estimation of swelling, atrophy, and midline shift

We estimated day 2 brain swelling and day 30 atrophy by calculating the ratio of ipsilesional and contralesional hemisphere volumes and the lateral displacement of the midline (Fig. 4B). To ensure consistent anatomical referencing, these calculations were performed in standard atlas space. We first located the anatomical midpoint based on ventricular geometry. The midline was defined by calculating the intersection of the subject’s segmented CSF mask with an atlas-defined midline restriction mask. The restriction mask isolated voxels from the lateral and third ventricles in a coronal section (seven voxels thick) located roughly at the midpoint of the corpus callosum in the anterior-posterior direction, estimated from the average 3D position of CSF.

The centroid of the CSF labels within this intersection was extracted and compared against standard anatomical landmarks. The lateral displacement of this centroid relative to the atlas midline defined the midline shift. When no midline CSF could be detected, such as in a case of severe cavitation or distortion, the algorithm handled the failure by reporting the shift metrics as not available (NA). We then defined a 3D quadratic surface that splits the left and right hemispheres based on this estimated midpoint. We created volumetric masks labeling the left and right hemispheres based on this surface (Fig. 4B, pink and yellow shades). We estimated several metrics, including midline shift (absolute, percent, and ratio), as well as tissue and brain volume laterality indices; explicit mathematical formulas for these metrics are documented in the supplementary GitHub repository. The accuracy of this automated midline estimate was manually validated by a trained anatomist for this entire cohort.

#### 2.5.8 Analytic reporting and network feedback

The final step in the pipeline created reports summarizing these outcome measures across the study cohort in data tables that were shared with the statistics team within the network periodically. These were used for providing biweekly feedback to sites about lesion volumes and whether they stay within pre-specified control limits over time. At the conclusion of each stage, MRI outcomes supported the Steering Committee’s decision-making process of determining which treatments meet efficacy or futility criteria.

## 3 Results

### 3.1 Sample sizes and network performance during the trial

Of the total 2,442 animals in the mITT cohort, 2,217 (91%) had an MRI on day 2, and 1,750 (72%) had an MRI on day 30. Mortality was the most common reason for missing MRIs. Only 24 animals died during day 2 MRI, of which 18 were aged mice; therefore, the contribution of performing an MRI under anesthesia on day 2 after 60-minute filament MCAO to the overall mortality appeared relatively small. An additional 79 animals died on day 2 after the MRI, but whether the MRI played a role was unclear. Equipment failure accounted for only 17 missing MRIs on day 2 (0.7%) and 3 missing MRIs on day 30 (0.1%). Most MRIs were successfully uploaded to the CC (99.8%). In total, 36 mouse scans (n = 18 day 2, n = 18 day 30) and 9 rat scans (n = 2 day 2, n = 7 day 30) failed the pipeline due to data quality issues (i.e., motion artifacts, incorrect FOV, field inhomogeneities) caused by technical hardware or scan protocol errors.

### 3.2 Brain extraction results

**Fig. 5.**
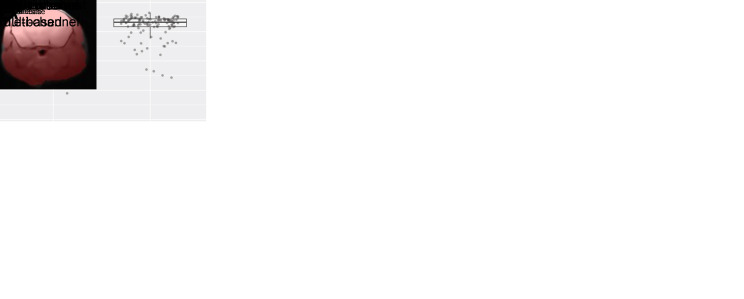
U-Net brain extraction performance in mice (left to right): (A) Comparison of traditional segmentation and M-full-multi (FM) U-Net on a fail and a training case; (B) Dice scores for (top left) FM vs full-single (FS) across all sites; (top right) FM vs FS broken down by half-sites; (bottom left) all four single contrast models (ADC_base_, ADC_rate_, R2_base_, R2_rate_); (bottom right) FS broken down by site.

The U-net models were performed with high accuracy across all test conditions. Our primary all-sites multi-channel model achieved the highest overall Dice score of 0.964 ± 0.028 (Fig. 5). The all-sites single-channel model had only slightly lower performance than the multi-channel model (Dice score 0.957 ± 0.033). Training the models on half (JH, IW, YL) of the sites and testing them on the other half (AU, MG, UT) resulted in mean Dice scores of 0.953 ± 0.026 (half-sites multi-channel) and 0.949 ± 0.040 (half-sites single-channel), indicating that both models can perform robustly on data from sites added to SPAN in the future. Looking at the data qualitatively, both the all-sites multi-channel and all-sites single-channel models had a 0% failure rate. Restricted models for individual contrasts also had robust Dice scores (all-sites ADC_base_ = 0.964 ± 0.026, all-sites ADC_rate_ = 0.958 ± 0.020, all-sites R2_base_ = 0.935 ± 0.104, and all-sites R2_rate_ = 0.954 ± 0.026), indicating that the model can be trained on a single contrast and still perform robustly on that contrast. When broken down by site, the Dice scores reflected image quality, with Site F having the highest SNR and the lowest Dice scores of 0.914 ± 0.040 and 0.919 ± 0.024 for the all-sites multi-channel and all-sites single-channel models, respectively. This site was the only site using a surface receive coil (all other sites had volume coils). In both all-sites multi-channel and all-sites single-channel models, performance was better with the late (day 30 after MCAO) than the early (day 2 after MCAO) time points (Table 2), likely because the late time point had less contrast heterogeneity and morphometric distortion than the early time point.

**Table 2.** Quantitative performance (Dice score) of U-net brain extraction models in mice.

		Individual sites						All sites by scan date	
Channels	All sites	AU	JH	MG	IW	UT	YL	Day 2	Day 30
Multi-channel	0.964	0.974	0.976	0.980	0.972	0.965	0.914	0.953	0.974
Single-channel	0.957	0.967	0.967	0.973	0.952	0.964	0.919	0.946	0.968

Johns Hopkins University (JH), University of Iowa (IW), Yale University (YL), Massachusetts General Hospital (MG), University of Texas McGovern Medical School at Houston (UT), and Augusta University (AU).

Lastly, we selected a group of 206 cases that failed catastrophically when processed by the traditional rule-based brain extraction approach, and we looked at the qualitative performance of the U-net in these specific cases. The all-sites multi-channel U-net model successfully extracted all of these cases. Two independent quality checks of the segmented data from the first stage of the SPAN study (n = 1,368 4-channel scans) showed a 4% failure rate on large multi-site preclinical mouse ADC and R2 datasets with ischemic stroke. Some of the cases that failed quality control were due to motion artifact, operator error, etc., and were excluded. Therefore, the model demonstrated excellent generalization to unseen sites and successfully segmented cases that caused traditional methods to fail, confirming its robustness for deployment in a multi-site network.

### 3.3 Lesion segmentation results

#### 3.3.1 Validation with manual tracing of MRI sections

We performed an initial validation experiment to determine the accuracy of our lesion segmentation. We first selected 10 typical day 2 scans, and 2 SPAN network stroke experts estimated lesion volume from manually delineated coronal sections of the R2_rate_ parameter map using ImageJ. All 10 cases were analyzed by both raters, and they were blinded to the results of our pipeline. The same 10 cases were subsequently processed with our pipeline without manual intervention to obtain the lesion volume for comparison in each case. We compared the human raters and the automated approach by computing descriptive statistics, the root-mean-square error (RMSE), and Pearson’s correlation coefficient. We found the average lesion volume from manual raters was 11.56 ± 8.56 mm^3^, and that from the automated approach was 11.38 ± 10.13 mm^3^. The RMSE between the two human raters was 2.22 mm^3^, and the RMSE error between the humans and the automated approach was 2.99 mm^3^. The Pearson correlation between the two human raters (R = 0.969; t = 11.17, df = 8, p-value = 3.7e-06, 95% CI = [0.871, 0.990]) was nearly identical to that between the human raters and the pipeline (R = 0.957 t = 9.338, df = 8, p-value = 1.412e-05, 95% CI = [0.823, 0.990]), with overlapping confidence intervals indicating statistically indistinguishable agreement.

#### 3.3.2 Validation with manual tracing of TTC-stained sections

**Fig. 6.**
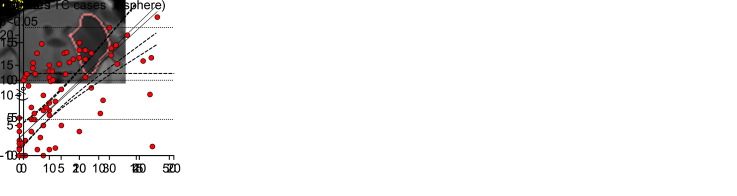
Validation of MRI measurement of infarct volume with tissue outcome assessment in the same mouse subjects. (A) The TTC-stained and R2 MRI coronal slices are shown from a representative mouse, with the infarct outlined in the latter by the automated pipeline. (B) Scatterplots show the correlation between TTC and MRI infarct volumes in the same animal with linear regression lines, 95% confidence intervals, and Pearson R. Left panel shows all cases, right panel shows only subjects with optimal TTC preparations. (C) Bland–Altman plot showing agreement between the two measures.

We estimated the reliability among human-TTC raters using the coefficient of variation (CoV = σ/μ) and measured Pearson’s correlation coefficient between the human-TTC and automated-MRI lesion volume fractions. We found human TTC raters to be highly reliable in delineating the whole brain (CoV = 2.50%); however, lesion segmentations had much greater variability (CoV = 18.5%). The TTC infarct volumes closely correlated with the MRI infarct volumes (Pearson R = 0.74; p < 0.001; Fig. 6B, left). We noticed that much of the discordance originated from poor interrater reliability in outlining TTC infarcts because of suboptimal brain slice preparation and staining (e.g., mechanical disruption or distortion of tissue, poor staining contrast). Therefore, we selected a subgroup of “high-reliability” TTC cases, as determined by human inter-rater lesion volume CoV < 5% (n = 25). The TTC infarcts in the high-reliability cases with optimal TTC slice preparation and staining showed an even stronger linear correlation with the MRI infarcts (Pearson R = 0.86; p < 0.001; Fig. 6B, right), validating our algorithm against the most commonly used method to delineate infarcts during the first few days after experimental stroke (Bederson et al., 1986). It is important to note that we did not attempt to correct for ischemic swelling, which leads to an overestimation of infarct volumes, especially *ex vivo* (Koch et al., 2019). This may have contributed to the slightly larger infarct volumes measured on TTC-stained slices compared with MRI in the same animal (slope = 0.77). Nevertheless, Bland–Altman analysis showed excellent agreement between the MRI and TTC infarct volumes in “high-reliability” TTC cases (bias 0.90, 95% limits of agreement, -5.20 to 7.00; Fig. 6C).

#### 3.3.3 Lesion segmentation performance

After automated segmentation, the distribution of harmonized R2_rate_ of the infarct (Fig. 4A, Day 2 R2_rate_ histogram, red line, and arrowhead) was clearly separated from both ipsilesional and contralesional non-infarcted tissue (Fig. 4A, Day 2 R2_rate_ histogram, blue lines, and arrowhead) with p < 0.001 for each using Kruskal–Wallis followed by Dwass, Steel, Critchlow-Fligner post hoc pairwise comparisons. The harmonized R2_rate_ within the ipsilesional and contralesional non-infarcted tissue differed only minimally, albeit significantly (p < 0.001; Fig. 4A, Day 2 R2_rate_ histogram, blue lines), suggesting that the increased water content (i.e., vasogenic edema) in the infarct did not infiltrate far into the surrounding tissue.

The harmonized ADC of the infarct (Fig. 4A, Day 2 ADC_rate_ histogram, red line, and arrow) was lower than that of both ipsilateral and contralateral normal tissue (p < 0.001 each); however, the distribution showed a modest overlap (Fig. 4A, Day 2 ADC_rate_ histogram, asterisk), precluding the ADC as a measure to segment the infarct. The harmonized ADC within the ipsilateral and contralateral normal tissue was nearly identical (p = 0.533; Fig. 4A, Day 2 ADC_rate_ histogram, blue arrow), suggesting that the segmentation based on the harmonized R2 threshold worked well and that all truly ADC-restricted voxels were captured within the segmented lesion.

**Fig. 7.**
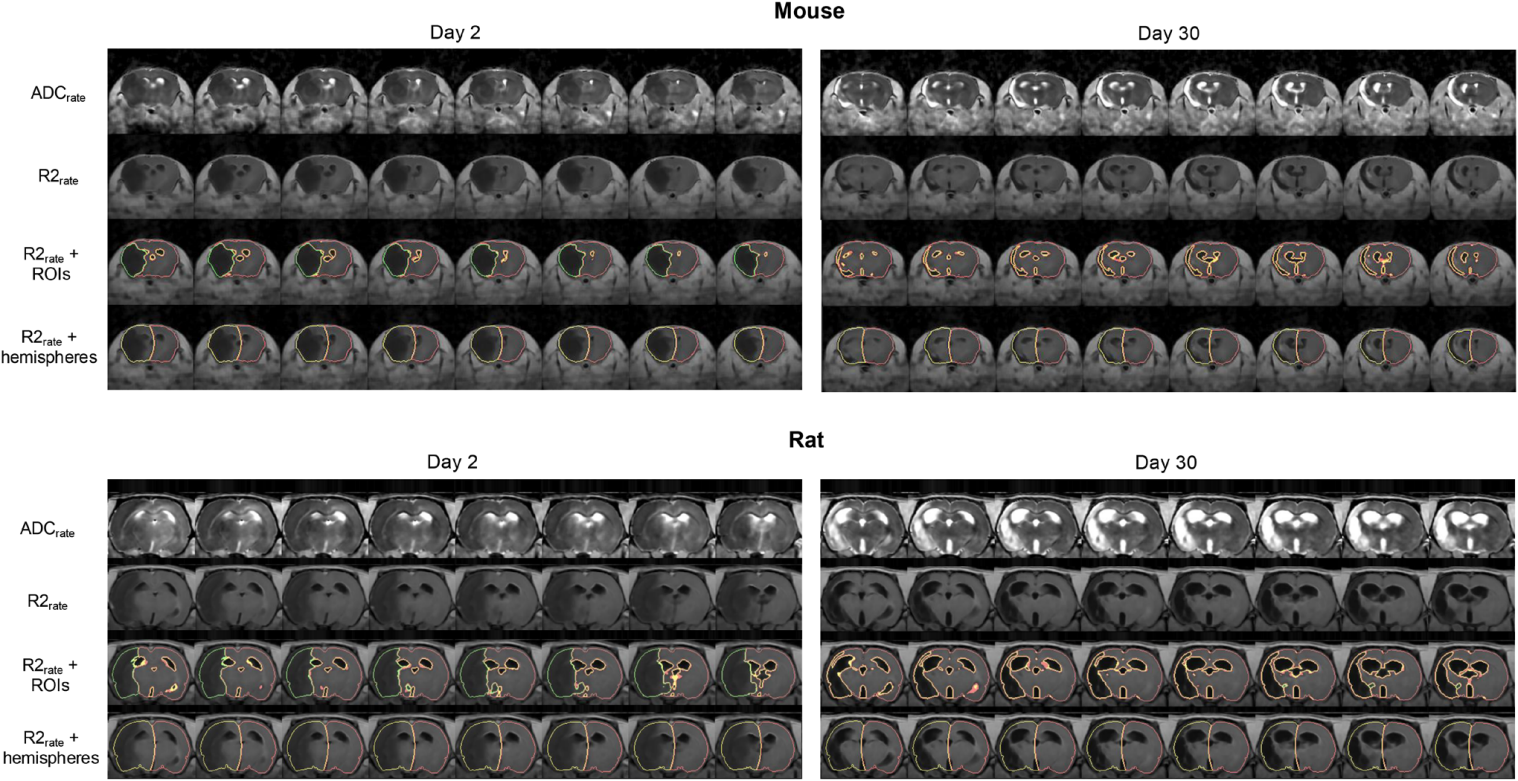
Example tissue segmentations and midline shift estimates in (top) mouse and (bottom) rat on (left) Day 2 after MCAO and (right) Day 30. Example ADC_rate_ and R2_rate_ data are shown for contiguous slices in an example mouse and rat on day 2 and 30 following MCAO. Tissue segmentations are superimposed on R2_rate_ data (R2_rate_ + ROIs) denoting outlines of normal-appearing tissue (pink), CSF (yellow), and lesion (green). Midline shift estimates are denoted by hemisphere segmentations superimposed on R2_rate_ data (R2_rate_ + hemispheres) showing the right hemisphere (yellow) and left hemisphere (pink).

Infarct volumes were quantified only on day 2 because day 30 scans showed atrophy and cavitation without clear lesion demarcation (Fig. 4B, Day 30 panel). Examples of the segmented infarct and CSF space on Day 2 and Day 30 in mice and rats are shown in Figure 7 and Supplementary Movies S1 and S2.

### 3.4 Midline detection performance

**Fig. 8.**
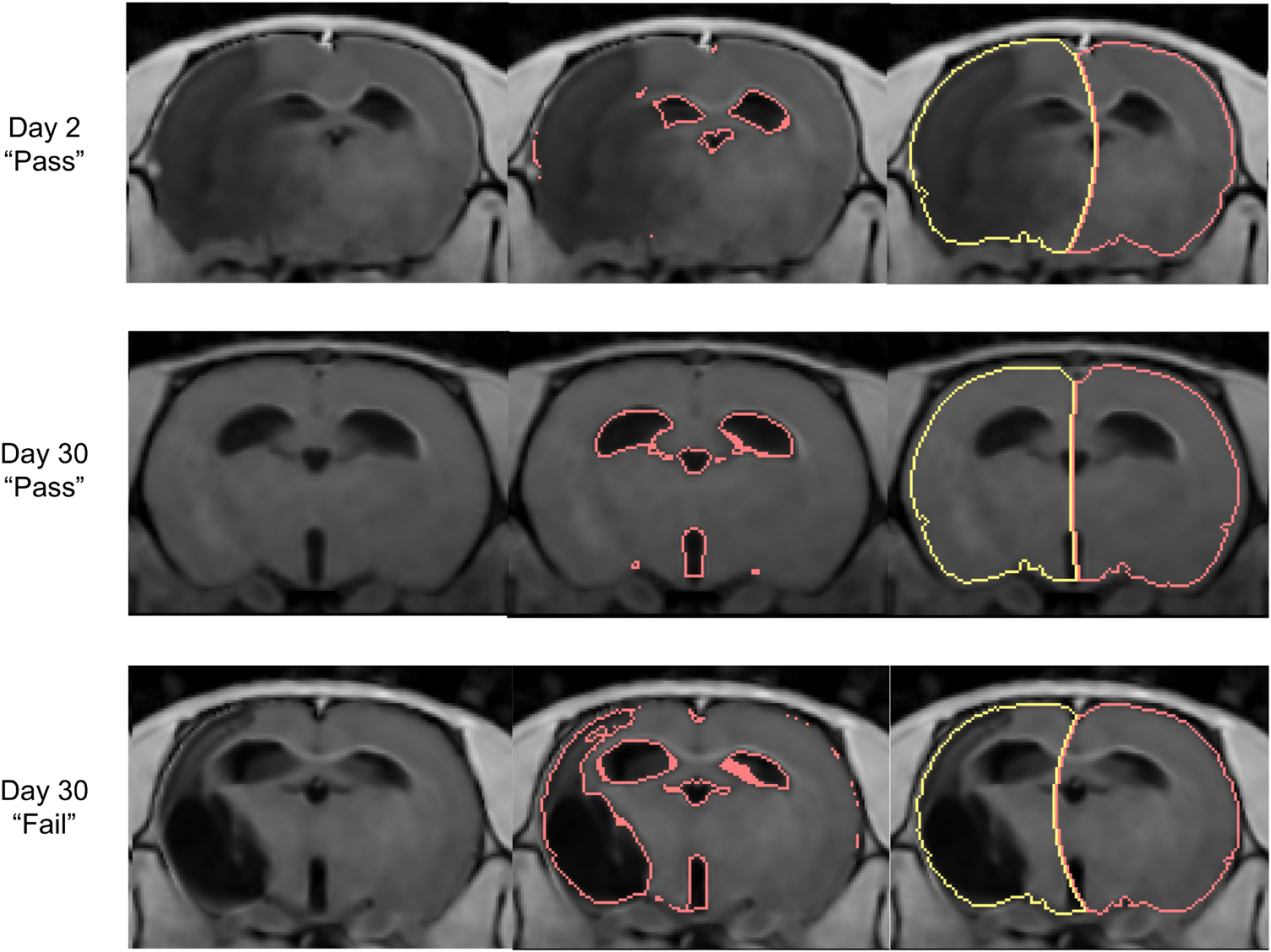
Automated midline shift estimation. Representative rat brains are shown from day 2 or 30 MRIs with the automatically drawn hemispheric and midline contours superimposed. The first two cases show successful midline estimation (i.e., “Pass”), and the third case shows a failed midline estimation (i.e., “Fail”) due to the presence of a large cavity, reflecting tissue loss upon severe infarction.

The accuracy of the midline estimate was ranked as “Pass” or “Fail” by a trained anatomist. In mice, all day 2 scans had acceptable midline estimates, while two day 30 scans (0.1%) had unacceptable midline estimates. In rats, 12 day 2 (2%) and 110 day 30 scans (20%) failed the midline shift assessment (Fig. 8). Upon further inspection, the worse performance observed in rats compared with mice was due to the presence of a large CSF compartment (i.e., cavitation) in the infarct bed on day 30. This CSF compartment significantly biased the centroid estimate intended to mark the third ventricle in rats. In contrast, mice displayed an ipsilesional midline shift rather than cavitation on day 30 scans; thus, midline estimation on day 30 was not affected in mice.

## 4 Discussion

In this study, we developed and validated a fully automated image analysis pipeline to address the critical needs of rigor, transparency, and reproducibility in preclinical stroke research. Applied to the large, six-center Stroke Preclinical Assessment Network (SPAN) trial with over 2,000 rodents, our method provided a robust, end-to-end solution for quantifying MRI-based tissue outcomes after experimental stroke. The primary contribution of this work is not just the successful analysis of an unprecedentedly large dataset, but also the creation of a durable and shareable framework that overcomes many inherent challenges of multi-site variability (Morais et al., 2023, 2024) and large-scale data processing that can be adopted by other multicenter preclinical stroke trial networks.

A fully automated pipeline was essential for the success of SPAN for three key reasons: scalability, objectivity, and reproducibility. The sheer volume of data, with tens of thousands of image slices, made robust manual delineation by researchers unfeasible. Automation was the only viable path to process the data in an effective and timely manner. Automation also removed the human inter- and intra-rater variability that plagues manual annotation, ensuring that all data are processed consistently and objectively, which is paramount in a blinded trial network. Finally, our automated pipeline is completely reproducible; by using version-controlled software and deterministic algorithms, we can ensure that results can be replicated by others, a core tenet of modern computational biomedical research.

While automated methods can sometimes be viewed as “black boxes,” we deliberately chose rule-based approaches where possible to maintain interpretability. Indeed, much of the design of the pipeline came from discussions of the network, allowing stroke and MRI domain experts to both understand and assist in the development process. For instance, our lesion segmentation uses a transparent thresholding method based on harmonized image parameters, which are readily understood by the entire team. Given the high accuracy of this approach against expert manual tracing (R = 0.96), transitioning to supervised learning for lesion segmentation is not currently a prioritized future direction. Training such models requires massive, manually delineated datasets, which introduces human inter-rater variability back into an automated process. While supervised learning may become necessary in the future for more complex tasks, such as differentiating subtle hemorrhagic transformations or secondary network degeneration, the transparent thresholding approach proved optimal and fully sufficient for our primary tissue outcomes. However, for brain extraction, our initial rule-based methods failed to achieve the required accuracy across all cases. We, therefore, developed a deep learning U-net model, which proved far more robust. By training and validating this model on a diverse, multi-site dataset, we established its reliability and confirmed its ability to generalize to data from unseen sites, suggesting it can be successfully adopted by other research groups. Our U-net model produces per-voxel softmax probabilities that are thresholded at 0.5 to yield the binary brain masks used in downstream analyses, following standard practice. Propagating these voxel-wise probabilities directly into group-level volumetric analyses, together with probability calibration and epistemic-uncertainty estimators such as Monte Carlo dropout or deep ensembles, represents a methodologically interesting direction for future work that could provide principled uncertainty bounds on derived tissue metrics.

The development of our pipeline involved several key decisions to handle the real-world complexities of multi-site data. A major challenge was the systematic difference in image contrast and intensity across scanners. We addressed this by implementing a simple yet effective harmonization procedure that scaled each image based on the mode of its intensity distribution, significantly reducing site-specific variability and allowing for consistent segmentation thresholds across the entire cohort.

Our choice of MRI parameters was also critical. We validated our initial hypothesis that R2 maps would be the most robust measure for delineating the infarct at day 2, while ADC maps would be most useful for differentiating the lesion from cerebrospinal fluid (CSF). While advanced sequences such as multi-directional diffusion tensor imaging (DTI) yield superior microstructural contrast, we deliberately utilized a rapid, single-direction DWI sequence with clinical standard b-values (up to 1,000 s/mm²). This approach intentionally approximated human acute stroke protocols while keeping total scan times strictly under 45 minutes, which was paramount for minimizing anesthesia exposure and maximizing survival in acutely ill rodents across a multi-center network. This aligns with previous meta-analyses showing that R2 is a reliable index of tissue damage several days after stroke (Milidonis et al., 2015), whereas ADC values can subsequently renormalize and show greater overlap with healthy tissue (H. An et al., 2011; Hsia et al., 2019). Our approach effectively uses the ADC map to serve a similar purpose as the fluid-attenuated inversion recovery (FLAIR) sequence in clinical MRI, which nulls the CSF signal to improve lesion conspicuity.

Another significant challenge was atlas registration. We found that the large, heterogeneous injuries and subsequent brain distortion from edema or atrophy made accurate non-linear registration to a standard atlas problematic. Rather than depending on a potentially flawed registration, we designed our analysis to be robust at the individual subject level. We used a simple affine registration only to standardize image orientation and provide coarse anatomical priors for locating the ventricles and the expected lesion hemisphere. This approach proved sufficient for our primary goals of quantifying total lesion and hemisphere volumes without being confounded by inaccurate warping.

This work has some limitations. First, while our automated pipeline successfully quantifies ischemic lesion volume, edema, and atrophy, it was not designed to detect or quantify hemorrhagic transformation. An early attempt to include a T2* sequence for this purpose proved unfeasible due to the low frequency of hemorrhage and significant signal artifacts. Future work could incorporate more advanced sequences to capture this important aspect of stroke pathology. Relatedly, our reliance on R2 maps at day 2 does not capture more subtle injuries, such as selective neuronal loss in moderately ischemic tissue or secondary atrophy from remote network disruption. More advanced techniques requiring higher-resolution imaging and multi-directional DTI (e.g., tractography) would be needed to fully characterize the downstream effects of the ischemic injury on brain networks. Third, our geometric, CSF-based method for midline estimation failed in a subset of rats at the late time point, where significant cavitation biased the calculation. Future work could explore more robust, template-based approaches to improve midline detection in cases of severe tissue loss. Fourth, recognizing that large, heterogeneous injuries make perfect non-linear atlas registration fundamentally unreliable, we deliberately designed our primary analyses to be robust at the individual subject level. However, future iterations could benefit from improved alignment. Developing a study-specific template that accounts for expected stroke-induced anatomical shifts, or using more advanced non-linear registration techniques, could allow for a more precise, region-based analysis of lesion distribution and its relationship to functional outcomes. Additionally, acquisition of baseline MRI scans prior to MCAO and subsequent brain pathology may aid in more precise atlas-based registration. Lastly, the pipeline design depends on rodent brain anatomy and a predictable location of the lesion in a standardized model—factors that might limit the translation to human stroke, where infarct locations and even numbers vary greatly.

## 5 Conclusion

We present a fully automated, open-source MRI analysis pipeline and protocol that successfully meets the demands of a large-scale, multicenter preclinical stroke trial. By addressing challenges of data heterogeneity, scale, and reproducibility, this work provides a validated framework for obtaining reliable, quantitative tissue outcomes from MRI data. The methodology enhances the rigor of preclinical research and offers a shareable resource that can be implemented by other research groups, ultimately establishing a foundation of highly reproducible data that is a necessary prerequisite for evaluating the true clinical potential of experimental stroke therapies.

## Supplementary Material

Supplemental Movie 1

Supplemental Movie 2

## Data Availability

MRI data used in the present study are available for download at: https://datadryad.org/stash/dataset/doi:10.5061/dryad.xd2547dpb. The automated MRI processing pipeline used in the present study is publicly available and can be downloaded at: https://github.com/cabeen/span-mri.
